# Supplementary material for: The Transverse Mechanical Axis of the Pelvis for Post-Operative Evaluation of Total Hip Arthroplasty
Source: Biomedicines. 2023 May 8;11(5):1397. doi: 10.3390/biomedicines11051397 (PMC10216800; doi:10.3390/biomedicines11051397)
Supplement: Supplementary file 1 [file biomedicines-11-01397-s001.zip › biomedicines-2265941-supplementary.pdf]

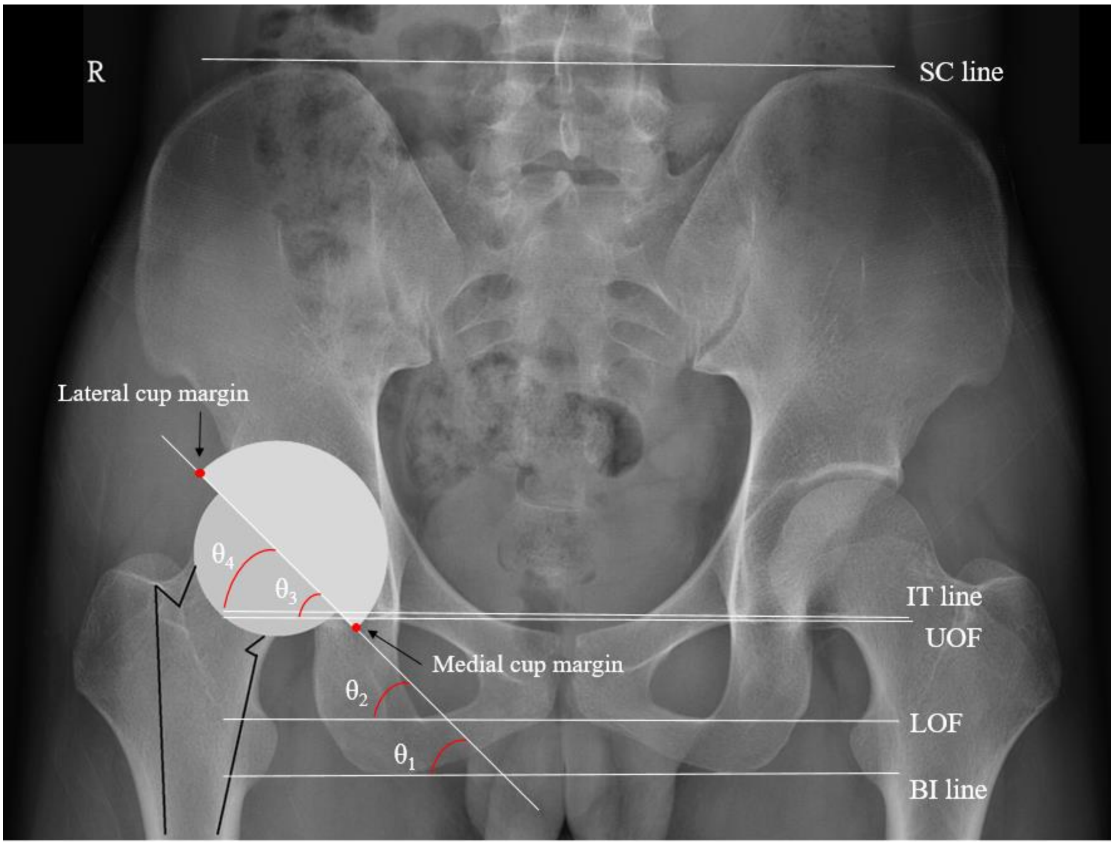

The above figure demonstrates the use of the five TAP measurements to determine the inclination angle of the acetabular implant. According to the definition, the inclination angle is the angle between the lines connecting the lateral and medial cup margins and the TAP. The angles labeled as θ_1 to θ_4 in the figure represent the inclination angle of the acetabular implant measured using the BI line, LOF, UOF, and IT line, respectively. Unfortunately, the SC line is located closer to the proximal end, making it challenging to draw the angle between it and the lines connecting the lateral and medial cup margins. Therefore, using the SC line to measure the inclination angle is not convenient on pelvic AP views.

As our IRB only enrolled participants without an acetabular implant at the time, the acetabular implant shown in the figure is a hand-drawn illustration.

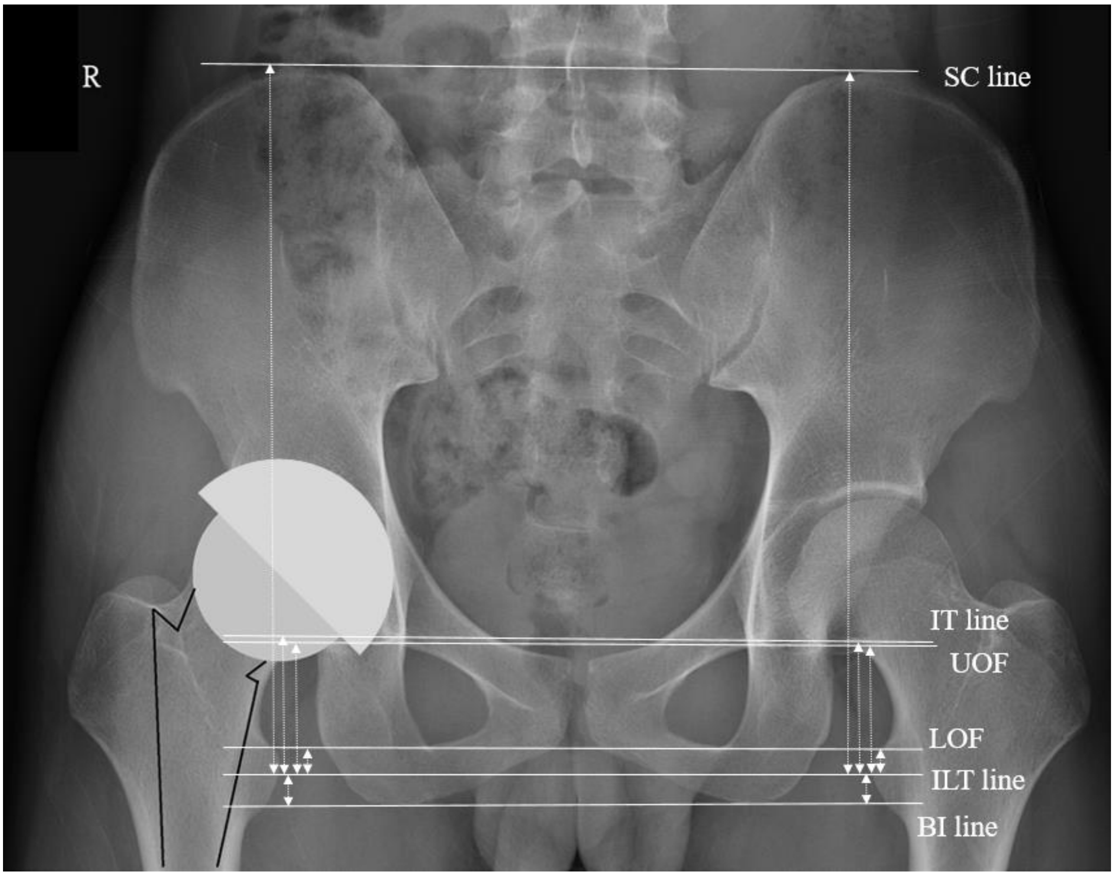

The above figure shows the LLD measurements obtained using the five TAPs after THA. According to the commonly used measurement method, LLD is the difference in vertical distance between the two sides of the inter-lesser trochanter line (ILT line) and the TAP.
